# Research on the Early-Warning Model of Network Public Opinion of Major Emergencies

**DOI:** 10.1109/ACCESS.2021.3066242

**Published:** 2021-03-17

**Authors:** Li-Jie Peng, Xi-Gao Shao, Wan-Ming Huang

**Affiliations:** School of Mathematics and Statistics ScienceLudong University 12405 Yantai 264025 China

**Keywords:** Major emergencies, internet public opinion, early warning index system, COVID-19, CRITIC, GA-BP neural network

## Abstract

The rapid development of Internet in recent years has led to a proliferation of social media networks as people who can gather online to share information, knowledge, and opinions. However, the network public opinion tends to generate strongly misleading and a large number of messages can cause shocks to the public once major emergencies appear. Therefore, we need to make correct prediction regarding and timely identify a potential crisis in the early warning of network public opinion. In view of this, this study fully considers the features of development and the propagation characteristics, so as to construct a network public opinion early warning index system that includes 4 first-level indicators and 13 second-level indicators. The weight of each indicator is calculated by the “CRITIC” method, so that the comprehensive evaluation value of each time point can be obtained and the early warning level of internet public opinion can be divided. Then, the Back Propagation neural network based on Genetic Algorithm (GA-BP) is used to establish a network public opinion early warning model. Finally, the major public health emergency, COVID-19 pandemic, is taken as a case for empirical analysis. The results show that by comparing with the traditional classification methods, such as BP neural network, decision tree, random forest, support vector machine and naive Bayes, GA-BP neural network has a higher accuracy rate for early warning of network public opinion. Consequently, the index system and early warning model constructed in this study have good feasibility and can provide references for related research on internet public opinion.

## I. Introduction

Major emergencies are often hard to predict, influence widely, with a complex situation, high sensitivity, and serious consequences [5]. Once mishandled, it will inevitably give rise to adverse reactions to the public and serious harm to society. In this case, the government needs to set out corresponding measures to deal with major emergencies. During the occurrence of major emergencies, people will express opinion and spread speech on the Internet. It is because the number of speakers on the Internet has greatly increased with the rapid development of network systems and the spread of information technology [15]. In the whole network environment, vital emergent events tend to enjoy a multitude of trigger points and relatively fast network propagation speed. Therefore, it is arduous to predict the system, law and path of the spread of public opinion. Information technology has given the number of new programming languages in which data mining and prediction can be effectively realized [9].

All kinds of network media and short video platforms emerge endlessly with the development of advanced mobile terminals and social media. In the Internet age, the network has an increasing influence on the behavior pattern, views, political attitudes, and emotional tendencies of individuals [21]. Therefore, explosiveness and instructiveness are the effect of the public on network public opinion in emergencies, which relate to the whole public opinion interaction network and even the direction of the interaction [17]. Unexpected incidents have tremendous and negative impact to folk’s life and spiritual level due to its own characteristics. More importantly, the severity level of the event is invariably proportional to the psychological impact on the public. Once errors occur in the dissemination of information, it is intensely easy to lead to panic in the public in serious cases. The loss of the ability to judge and analyze information can well possess nasty ramifications in society. If it is not emphasized to create a harmonious network environment, the network public opinion will have a great negative impact on the society in the whole communication process.

The calculation and evaluation of average inclinations to any opinion/sentiment toward any entity helps both the organization and the individual to get the right opinion about the ongoing trends or unfamiliar things [6]. At present, a slice of scholars try to study early warning of network public opinion from different perspectives. The research on early warning of internet public opinion is becoming a hotspot among scholars in public opinion research, which has captured extensive attention to the academic world. Scholars at home and abroad have studied network public opinion early warning from different aspects such as fuzzy comprehensive method, intuitionistic fuzzy reasoning, Bayesian network, BP neural network, support vector machine, and so on (e.g., [8], [22]). The main research is the early warning research of network public opinion with “the index system + evaluation model” mode [11]. That is based on the scientific and reasonable methods to determine the index dimension, the specific meaning of each index and the quantitative method. And thus, the early warning index system of internet public opinion is established. Then the modeling approach is selected to determine the warning level. The study of network public opinion pre-warning in China starts late. But with the development of internet technology and mathematical models, it has reached a new developmental stage. Wang *et al.* [19] put forward the fuzzy comprehensive evaluation method to the research of network public opinion prediction. They used the appropriate network public opinion early warning system to determine the weight of each index and predict the warning level. Cao *et al.* [2] used the analytic hierarchy process and the fuzzy comprehensive evaluation method to construct the early warning level model of public opinion related to the old network. However, public opinion emergencies generally possess randomness and uncertainty. The models described above cannot handle the non-linear relationship between input and output well. Fortunately, the Artificial neural network can be used to deal with strong non-linear processing. It is designed to be highly fault-tolerant and adaptive capacity. Zhao and Pan [26] built a network public opinion early warning index system, and the BP neural network is used to establish a network public opinion early warning model to give an early warning level of network public opinion events. Their results demonstrated that public warning accuracy has been improved and needs to be further improved.

The key to achieving internet public opinion pre-warning lies in establishing an assessment index system and a prediction model. However, there are many problems about the early warning of internet public opinion. Most existing research approaches focus on theoretical research and have a lot of repetitiveness. Too much emphasis is placed on the interpretability of the index system, which tends to achieve poor predictive accuracy. The innovative technology of internet public opinion pre-warning needs to be further strengthened. To establish a scientific and reasonable early warning index system of the internet public opinion, it is necessary to consider both the early warning level scores and prediction methods. The majority of the existing studies have investigated the public opinion early warning level using the composite weight score and experts’ marking [16], [26]. However, this type of approach tends to harbor a degree of subjectivity and lacks of the originality of the scientific. For this reason, we apply a more objective, scientifically rigorous, and replicable approach, the “CRITIC” method. This method has high sensitivity and simple principle. It not only takes into account the influence of index variation degree on the weight, but also considers the impact of the conflict between early warning indicators on the weight. In terms of prediction for internet public opinion early warning, as is known, BP network has the characteristics of self-organization, self-adaptation and self-learning. However, this model is very sensitive to the initialization and easily getting into the local minimum, which limits its practical applications. In light of these questions, GA-BP, a hybrid artificial intelligent algorithm with genetic algorithm and Back-Propagation (BP) neural network, may be proposed to optimize it. Many researches have been carried out to the GA-BP algorithm, but there is a dearth of such research in the early warning of the network public opinion.

This study fully considers the transmission rules, the propagation characteristics and the developing trend of public opinion. As a result, the mechanism of the network public opinion early warning is constructed. Moreover, this research analyzes the indicators that enjoy a significantly crucial impact in the process of generating and spreading network public opinion and establishes the network public opinion early warning index system. Furthermore, the CRITIC method [3] is used to classify the early warning level of the network public opinion. Finally, combining Genetic Algorithms (GA) and back-propagation neural network (BP), an GA-BP model (e.g., [4], [17], [13], [23]) is established to predict the level of the network public opinion. The internet public opinion data of COVID-19 pandemic after processing are chosen to train the network model. Comparing the results from traditional BP network, decision tree, random forest, naive Bayes and support vector machine for forecasting the level of the network public opinion, the optimal warning model is determined. In this study, the purpose is to explore the application value of the network public opinion warning model in the case of major emergencies. This will help managers to put forward reasonable internet public opinion supervision strategies and implementation plans and to point out its existent latent crisis in time.

Given the above, the present paper has the following structure. Based on the advantages and disadvantages of the existing index system, the network public opinion warning index system for major emergencies is established and the meaning of each index is explained in Section 2. For background, the CRITIC evaluation methodology and GA-BP neural network are overviewed in Section 3. In Section 4, taking major public health emergency COVID-19 pandemic as an example, the early warning level of internet public opinion is determined by the CRITIC method and predicted by the GA-BP neural network. Besides, other familiar machine learning approaches have been compared and analyzed. Finally, a summary of the paper results and a further understanding are considered in Section 5.

## II. Build the Network Public Opinion Early Warning Index System

A reasonable index system is the guarantee of effective early warning, so it is indispensable to establish a major emergency network public opinion warning index system composed of relevant indicators [11]. The early warning index system of network public opinion for major emergencies can not only reflects the current situation and existing problems of internet public opinion but also promptly provides early warning for emergencies [10]. The indicators in the index system are the basis and the criteria for evaluating the seriousness of network public opinion. The index system is useful to study the network public opinion and classify its risk levels in major emergencies. The determination of index items will directly affects the accuracy and comprehensiveness of the early warning level. In the process of building the indicator system, the six principles listed in Table 1 should be followed.

**TABLE 1:** Six Principles of Building the Network Public Opinion Early Warning Index System

Principle	Description
Principle of profession	The network public opinion indicators involve many disciplines. The selection of indicators should conform to the workflow of public opinion early warning, and in principle, should be consistent with the research topic or major.
Principle of quantification	When selecting indexes, we should try to choose indexes that can be quantified and described objectively.
Principle of practicality	In the theoretical sense, internet public opinion indicators are often arduous to define. In the selection of indicators, it is indispensable to consider the operability of indicators, which cannot be limited within the theoretical scope to make it infeasible to quantify the indicators.
Principle of importance	The network public opinion covers a wide range. Therefore, there is generally an ocean of influencing factors related to public opinion. If the comprehensiveness of early warning indicators is considered, the redundancy will be high and will inevitably lead to the overlapping of indicators. Therefore, the main influencing index are selected.
Principle of economy	The selection of indicators should be within a certain range of acquisition ability, so as to avoid paying a great price in the face of great difficulty in obtaining indicators.
Principle of sensitive	It is required that the index should enjoy good sensitivity, and can demonstrate the change trend or value according to the development degree of the event in time, so as to avoid the selection of the warning index with underdeveloped sensitivity.

This study fully considers the laws and changes in the process of network public opinion transmission when major emergencies appear. Further, through research and consultation with relevant experts, we eventually establish a network public opinion warning index system for major emergencies with 4 first-level indicators and 13 second-level indicators after repeated rectification and selection. All the indicators in the index system are within the acquired capability and meet the above construction principles. Importantly, all of these indicators can be quantified. The early warning index system of network public opinion for major emergencies is demonstrated in Table 2.

**TABLE 2:** Network Public Opinion Warning Index System for Major Emergencies

First-level	Second-level
Attention	Search
	Media
	Media release
Participation	Original tweet
	Comment and retweet
	Thumb up
	User influence
Diffusion	Change in search
	Change in original tweet
	Change in comment and retweet
	Change in thumb up
Status	Visual
	Authenticity

The data for these indicators comes from Sina Weibo and Baidu index. Sina Weibo is one of the largest information transmission and public social networking platforms, with a large number of network users [8]. Sina Weibo is a Chinese microblogging (weibo) website and enjoys a strong ability for instant interaction. Its users are not only the receivers of information but also the publishers and disseminators of information. The openness and rapid dissemination of Weibo can quickly turn viewpoint into network public opinion [12]. Baidu is one of the largest search engines. It offers the Baidu index dedicated to finding the number of specific topics and posts. Therefore, it is representative and reasonable to build a network public opinion warning index system for major emergencies by combining relevant contents from Weibo and Baidu index.

As can be seen from Table 2, the early warning index system of network public opinion for major emergencies is divided into two levels. The first-level index system mainly includes four dimensions: Attention, Participation, Diffusion, and Status. The Attention dimension refers to the attention of netizen and media to public opinion topics, which is measured by the search volume of netizens and volume of media reports. The Participation dimension represents the degree of participation of netizens in the discussion of public opinion topics, and it is measured by the number of “posts”, “comments”, “reposts” and “like”, etc. The Diffusion dimension characterizes the diffusion trend of public opinion in the transmission process, which is explained by the degree of change in the value of certain indicators. The Status dimension describes a slice of nature of the public opinion itself. A detailed description of the second level indicators is given below.

The Attention dimension consists of three second-level indexes. The Search represents the number of times that netizens search public opinion topics. It is indicative that the degree of exposure of the public opinion event in a specific period. The Media refers to the number of media participating in reporting public opinion topics in 15 mainstream media. The Media release reflects the number of reports published by the Baidu on all internet media platforms.

The Participation dimension includes four second-level indexes. The Original tweet is the number of original tweets. The Comment and retweet reflect the number of retweets and comments on tweets related to the topic of public opinion. The Thumb up refers to the number of times that tweets related to the public opinion topic are liked by other users. It reflects the content appeal of tweets or the interactive influence of bloggers. Also, the User influence is quantified by multiplying the number of V bloggers among users who have published posts related to the public opinion topic by 0.8 plus ordinary users by 0.2.

The Diffusion dimension contains four second-level indexes. They are used to describe the change of the above part of indexes. Taking the Change in search as an example, assuming that the Search at time P1 is R1 and the Search at time P2 is R2, the Change in search from time P1 to time P2 is (R2-R1)/R1. The other three second-level indexes are also defined in this way. For this reason, they are not reported here.

The Status dimension consists of two second-level indexes. The Visual describes the proportion of the number of tweets posted by users in the form of pictures and videos related to the public opinion topic in the total number of tweets. The Authenticity refers to the ratio of the number of public opinion related tweets posted by users with real-name authentication to the total number of published tweets.

## III. Research Methods

### A. Critic Method

The full name of CRITIC is Criteria Importance Though Intercrieria Correlation, which is an objective weight method proposed by [3]. The method is superior to usual the entropy weight method and the standard deviation method. The objective weights of indicators determined by CRITIC are based on two basic concepts: indicator variability and indicator conflict [1].

The indicator variability refers to the difference between the evaluation observations of the same indicator, which is expressed in the form of standard deviation. The indicator conflict is based on the correlation between indexes. If there is a high positive correlation between indexes, it is suggestive that the conflict between indexes is small and the weight of this index is small. Assuming that there are $i$ samples and $j$ evaluation indexes, the original data matrix can be expressed as:

\begin{align*} X=\left ({\begin{array}{llll} x_{11}&\quad \cdots &\quad x_{1j}\\ \vdots &\quad \ddots &\quad \vdots \\ x_{i1}&\quad \cdots &\quad x_{ij} \end{array}}\right)\tag{1}\end{align*}

where $x_{ij}$ represents the $j$th index value of the $i$th sample. In general, the dimension of each index is different. In order to unify dimensions and make data comparable, it is generally indispensable to standardize or normalize the original data. However, the use of standardization is not recommended here. The reason is that the standard deviation of all indicators will become 1 after the standardization. There is no doubt that using the CRITIC method in this case will be meaningless. Therefore, to make rational use of the CRITIC method and distinguish between positive and negative indicators, the normalization method is chosen. If the indicator has a positive polarity:

\begin{equation*} x_{ij}=\frac {x_{ij}-min{(x_{ij})}}{max{(x_{ij})}-min{(x_{ij})}}\tag{2}\end{equation*}

If the indicator has a positive polarity:

\begin{equation*} x_{ij}=\frac {max{(x_{ij})}-x_{ij}}{max{(x_{ij})}-min{(x_{ij})}}\tag{3}\end{equation*}

After the influence of dimension on evaluation result is eliminated, index variability and index conflict can be expressed respectively. The indicator variability is the standard deviation of each indicator:

\begin{align*} \begin{cases} \displaystyle \bar {x}_{j}=\frac {1}{n}\sum _{i=1}^{n}x_{ij}\\ \displaystyle S_{j}=\sqrt {\frac {1}{n-1}\sum _{i=1}^{n}\left ({x_{ij}-\bar {x}_{j}}\right)^{2}} \end{cases}\tag{4}\end{align*}

The indicator conflict is constructed by the correlation coefficient between the indexes:

\begin{equation*} R_{j}=\sum _{k=1}^{p}(1-r_{jk})\tag{5}\end{equation*}

where $r_{jk}$ represents the correlation coefficient between the $j$th index and the $k$ th index. The information volume of each index can be calculated by combining the index variability and index conflict:

\begin{equation*} C_{j}=S_{j}\times R_{j}\tag{6}\end{equation*}

Finally, the weight of each index is determined by the amount of information volume:

\begin{equation*} W_{j}=\frac {C_{j}}{\sum \limits _{j=1}^{p}C_{j}}\tag{7}\end{equation*}

Based on the index weight, the comprehensive evaluation index of each sample can be calculated, and then the samples can be graded. The specific analysis process is manifested in the empirical analysis section.

### B. GA-BP Neural Network Algorithm

**FIGURE 1.**
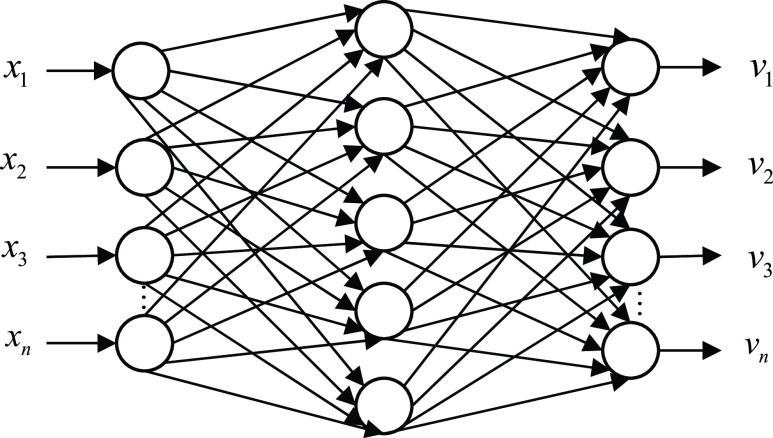
Structure of BP neural network.

BP neural network algorithm is a multi-layer feedback forward neural network structure, which is trained according to the backward error propagation algorithm [5]. This machine learning algorithm is implemented by simulating the brain. The BP neural network is composed of three layers, namely the input layer, the hidden layer, and the output layer. It can train and accommodate the mapping relation of the input-output model internally without reveal mathematical equations in advance to explain the mapping. The structure diagram can be seen in Figure 1. The array $x_{1},x_{2} \cdots,x_{n} $ represents the input layer neuron. $w_{ij} $ is the connection strength between the input layer and the hidden layer. $\mu _{jk} $ is the connection strength between the hidden layer and the output layer. $\theta _{j} $ is the node threshold of the hidden layer. $\gamma _{k} $ is the node threshold of the output layer. $f$ is the activation function of the hidden layer. $\varphi $ is the activation function of the output layer. $I_{j} $ is the net input value of neuron $j$.

\begin{equation*} I_{j} =\sum \limits _{i} {w_{ij} x_{i} +\theta _{j}}\tag{8}\end{equation*}

After the net input $I_{j} $ passes the activation function $f$, $m_{j} $ as the output of neuron $j$.

\begin{equation*} m_{j} =f\left({\sum \limits _{i} {w_{ij} x_{i} +\theta _{j}} }\right)=f(I_{j})\tag{9}\end{equation*}

Here, $f$ occupies some properties of monotonic function, namely, monotonic and limited. Because it is absolutely infeasible for a signal transported by the cell to increase extremely without reaching a maximum. $I_{s} $ is the net input of the output layer, $y_{k} $ is the output of the output layer.

\begin{align*} I_{s}=&\sum \limits _{j} {\mu _{jk} m_{j} +\gamma _{k}} \tag{10}\\ y_{k}=&\varphi \left({\sum \limits _{j} {\mu _{jk} m_{j} +\gamma _{k}} }\right)=\varphi (I_{s})\tag{11}\end{align*}

**FIGURE 2.**
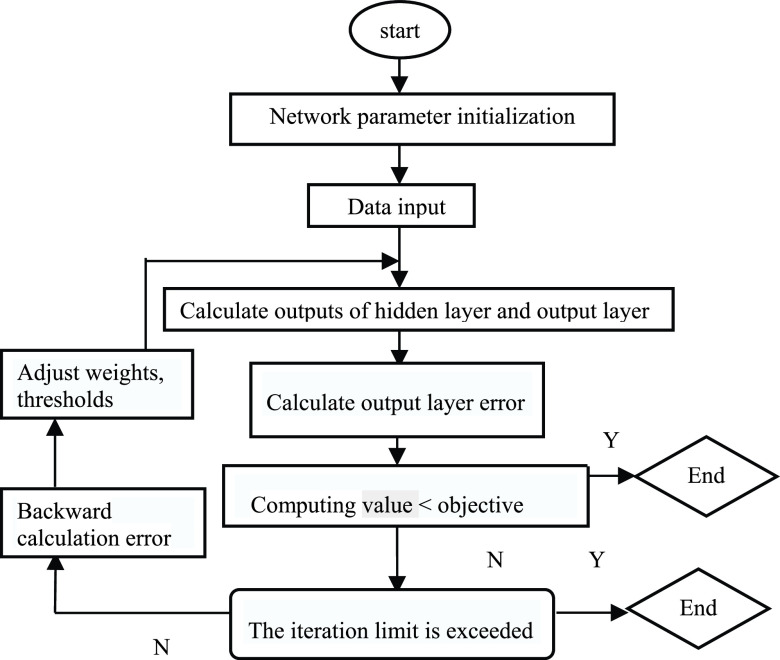
The process of the BP neural network algorithm.

The training process of BP neural network consists of error back propagation process and input data forward propagation process. The two processes constitute an iteration, the iteration stops when the prediction accuracy or target requirement are reached, and then the training process ends [25]. Forward propagation is to make the data input to the output layer by the corresponding weights and thresholds. When the calculation error has not met the set target, it will return and continue the backpropagation process. During the return process, the weights and thresholds of the hidden layer and the input layer need to be adjusted continuously. After that, the iteration continues until the error meets the requirement and the expected aim is obtained. The process of the BP algorithm is demonstrated in Figure 2.

**FIGURE 3.**
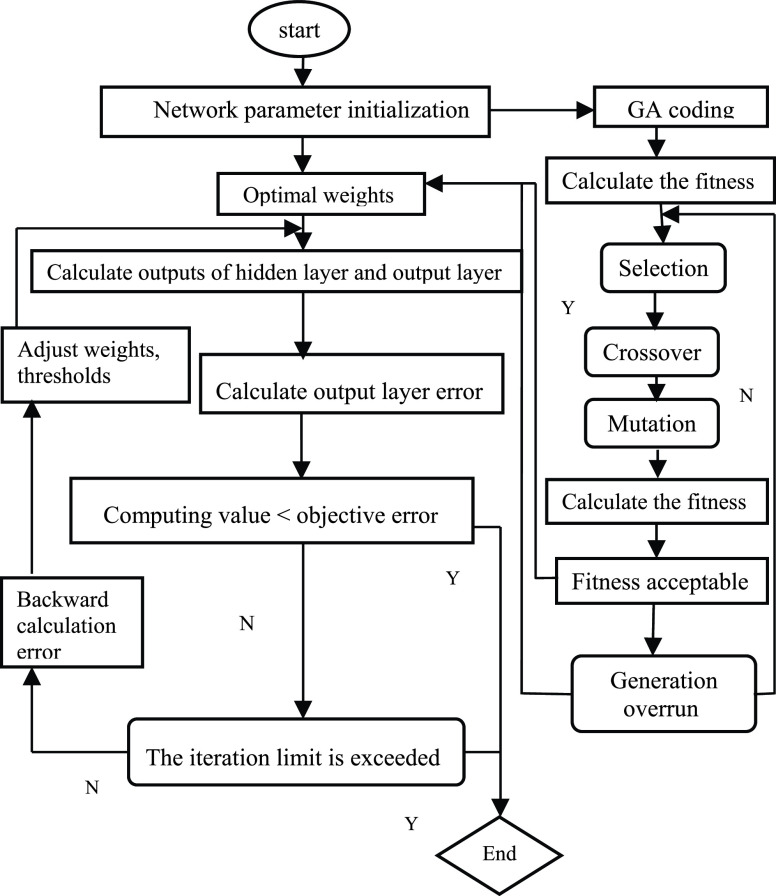
The process of GA-BP algorithm.

However, as noted previously, researchers point out the BP neural network still has quite a few shortcomings, such as it is affected by the value of the initial weight. In addition, BP neural network converges slowly and easily falls into the local minimum. Therefore, we need to adopt some optimization algorithms to overcome the shortcomings of BP neural network. At present, the global search optimal feature of GA can be used to make up for the deficiency of BP neural network algorithm. The process of the GA-BP algorithm is demonstrated in Figure 3.

GA is a computational model that simulates the genetic mechanisms of natural selection and biological evolution. It’s a way to search the optimization scheme by simulating natural evolutionary processes [24], [20]. GA is a global optimization search algorithm. The main steps of GA include encoding variables, generating initial set or initial solution space, allocating fitness value, replication, crossover, mutation, iteration until the end of the training. The algorithm conforms to Darwin’s theory of “survival of the fittest”. N represents the number of initial solution spaces, which are randomly generated after the encoding method is determined. The purpose of these steps is to form individual chromosomes with the highest degree of adaptation. The value will be used as the initial weight of the BP neural network for further learning and training. This way can decrease the impact on the initial weight of BP neural network. In this paper, GA-BP neural network algorithm is used in forecasting level of network public opinion. The training process of GA-BP neural network includes the following steps:

(1) The solution of the studied problem is represented by a code string, where each code represents a solution.
(2) An initial population is randomly generated, which is the initial solution space of the research problem.
(3) Convert the encoding string to an optimization parameter. The initial population fitness value is calculated according to the coded objective function.
(4) According to the value of fitness, replication, crossover and variation are carried out in turn. The purpose of this is to find the optimal individual.
(5) Return to steps 3 and 4 until the termination requirement is met. The previously coded individuals continuously evolve to obtain the optimal solution of the research problem, which is the initial weights and thresholds of the BP neural network optimized by genetic algorithm.
(6) The initial weights and thresholds are introduced into the BP neural network model for training until the upper limit of training times or error requirement are reached.

## IV. Empirical Analysis

COVID-19 is the disease caused by the new coronavirus that appeared in December 2019 in China. It is an illness caused by a virus that can spread from person to person. While health officials are still tracing the exact origin of the new coronavirus, an early hypothesis suggests that it may be related to a seafood market in Wuhan, China. According to a study that came out on Jan. 25, 2020, notes that the first reported case occurred on Dec. 1, 2019 and was not link to the seafood market. Investigations are under way into how the virus originated and spread. Symptoms of COVID-19 include cough, fever or chills, diarrhea, headache, shortness of breath or difficulty breathing, nausea or vomiting and so on. COVID-19 has come with a vengeance, and the number of new confirmed cases and deaths continues to rise.

During the outbreak in China, public opinion topics related to COVID-19 were closely followed. The development and transmission of internet public opinion reflect the prevention and control of COVID-19 from one side and demonstrate the degree of public concern over the incident. Therefore, this study selects major public health emergency COVID-19 as a case study of network public opinion events. The main progress of the event is manifested in Table 3 as of 30 April 2020.

**TABLE 3:** Progress on the Major Public Health Emergency COVID-19 in China

Date	Progress of the COVID-19 incident
2019/12/08	The earliest laboratory confirmed case of COVID-19 appeared in Wuhan, Hubei Province.
2019/12/30	Wuhan Municipal Health Commission reported a cluster of cases of pneumonia in Wuhan city, Hubei Province. Eventually, a novel coronavirus was identified.
2020/01/09	The first death from COVID-19 occurred in Wuhan.
2020/01/21	Confirmed cases have been reported in Tianjin, Zhejiang, Jiangxi, Shandong, Henan, Hunan, Chongqing, and Sichuan provinces.
2020/01/23	The central government of China imposed a lockdown in Wuhan.
2020/01/24	More than 1000 cases of coronavirus infection have been confirmed in China.
2020/01/28	The number of confirmed cases in China has surpassed the SARS virus that was circulating during 2002–2003.
2020/01/29	There are 1,737 new confirmed cases were reported by 31 provinces, autonomous regions and municipalities and the Xinjiang Production and Construction Corps.
2020/02/11	The World Health Organization (WHO) has officially named the coronavirus as COVID-19.
2020/02/16	The National Health Commission said the achievements were made in the epidemic prevention and control efforts across the country.
2020/02//19	Eight province-level regions, including Shanghai, in Chinese mainland reported zero new infections on Tuesday, Feb 18. The other regions are Hainan, Qinghai, Guizhou, Tibet, Xinjiang, Liaoning and Gansu.
2020/02/28	For the first time, the cumulative number of cured cases exceeded the number of confirmed cases.
2020/02/29	The total number of confirmed COVID-19 cases in Italy rises to 889, including 21 fatalities and 46 recoveries.
2020/03/04	The response level of 21 provinces was lowered, and only 5 new confirmed cases outside Hubei province.
2020/03/10	All 16 makeshift hospitals in Wuhan have been closed
2020/03/13	COVID-19 Solidarity Response Fund launched to receive donations from private individuals, corporations and institutions.
2020/04/08	China has ended its lockdown of Wuhan.
2020/04/11	New Coronavirus inactivated vaccine has been approved for clinical trials

### A. Data Acquisition and Preprocessing

In the construction of the network public opinion early warning index system for major emergencies, the data of the second-level index has been introduced from the Weibo platform and Baidu index. Most of the second-level indexes data can be directly obtained through Weibo advanced search and Baidu Index or calculated by other second-level indexes. However, a small part of the second-level indexes data can only be obtained through web crawlers, such as Comment and retweet, Thumb up, and User influence. The reason is that the data of these indicators need to be summarized by traversing all the tweets. The Weibo platform does not provide a summary of the information. Fortunately, [7] shared his weibo-public-opinion-datasets on GitHub. Here, the construction method of Weibo-public-opinion-datasets is briefly introduced.

The first step is to build and dynamically maintain a highly confidential pool of active Weibo users, which accounts for only a small percentage of all users. For the construction of an active Weibo user pool, a pool containing 250 million Weibo users is first established, and then the active Weibo user pool is screened out according to the four rules. The filtering rules are demonstrated in Table 4. As a result, a pool of active Weibo users with 20 million users is established, and its users accounted for 8% of the total number of Weibo users.

**TABLE 4:** Filter Rules for Active Users

Item	Rule	Item	Rule
Follows number	>50	Tweets number	>50
Fans number	>50	Recent post	<30 days

The second step is to use software built-in in Python language to crawl the tweets related to the COVID-19 topic posted by these 20 million active users within a specified time. As a result, the weibo-public-opinion datasets is established. This study can filter and extract the corresponding second-level index data from the data set. The data sources and properties of the second-level index are shown in Table 5.

**TABLE 5:** The Data Sources and Properties of the Second-Level Index

Variables	Second-level	Data sources	Properties
X1	Search	Baidu index	Positive; Quantitative
X2	Media	Weibo advanced search	Positive; Quantitative
X3	Media release	Baidu index	Positive; Quantitative
X4	Original tweets	Weibo advanced search	Positive; Quantitative
X5	Comment and retweet	weibo-public-opinion-datasets	Positive; Quantitative
X6	Thumb up	weibo-public-opinion-datasets	Positive; Quantitative
X7	User influence	weibo-public-opinion-datasets	Positive; Quantitative
X8	Change in Search	another second-level index	Positive; Quantitative
X9	Change in Original tweets	another second-level index	Positive; Quantitative
X10	Change in Comment and retweet	another second-level index	Positive; Quantitative
X11	Change in Thumb up	another second-level index	Positive; Quantitative
X12	Visual	Weibo advanced search	Positive; Quantitative
X13	Authenticity	Weibo advanced search	Positive; Quantitative

Based on the progress on the major public health emergency COVID-19 in China and the current relatively complete epidemic timeline published by Southern Metropolis Daily, this study preliminarily selects and collects network public opinion data of 55 critical epidemic time points between December 1, 2019, and April 31, 2020. After sorting out the data, it is found that all the index data of the selected time node are 0 except a few between December 1, 2020, and January 20, 2020. They are eliminated because they have no analytical value. The reason is that COVID-19 is not capturing enough attention from netizens at this time. In fact, the real outbreak of COVID-19 related internet public opinion starts with the lockdown of Wuhan city. Therefore, 45 paramount epidemic time nodes are selected as experimental data in this study between January 21, 2020, and April 30, 2020. Due to the large magnitude difference between the indicators, the data of each indicator is normalized according to formula (2) or formula (3) for the convenience of subsequent research.

The whole empirical analysis process is performed in the publicly available statistical software built-in in R language (R Core Team, 2019). The code and data are available in the supplemental materials.

### B. Setting the Early Warning Level

In order to give early warning to the level of COVID-19 internet public opinion, the level of internet public opinion is divided. According to the CRITIC method introduced earlier, the weight $W_{j }$ ($j=1, 2,\ldots, 13$) of each index is calculated, as demonstrated in Table 6.

**TABLE 6:** Weight of Each Warning Index

Index	X1	X2	X3	X4	X5	X6	X7
Weight	0.110	0.066	0.095	0.087	0.053	0.075	0.065
Index	X8	X9	X10	X11	X12	X13	
Weight	0.080	0.066	0.072	0.070	0.104	0.058	

Based on the weight of each index, the Comprehensive Evaluation Index (CEI) of each time node can be calculated. The CEI of each time node is equal to the sum of the evaluation indexes of each index at the time node. As an example, the CEI of the $i$ th time node is:

\begin{equation*} V_{i} =\mathop \sum \limits _{j=1}^{13} X_{ij}^{'} w_{\textrm {j}}\tag{12}\end{equation*}

where $X_{ij} $ represents the value of the $j$th index after normalization at the $i$ th time node; $X_{ij}^{'} w_{\textrm {j}} $ represents the evaluation index of the $j$th index at the $i$th time node.

As a result, the CEI for each time node can be obtained. To facilitate the classification of public opinion levels, the calculated CEI is appropriately transformed to make its range between 0 and 100. The transformation formula is as follows:

\begin{equation*} V_{i}^{\prime } =\frac {\max \left ({{V_{i}} }\right)-V_{i}}{\max \left ({{V_{i}} }\right)-\textrm {min}(V_{i})}\times 100\tag{13}\end{equation*}

The General National Emergency Response Plan for Public Emergencies issued by the State Council divides the early warning into level 1 (extremely severe), level 2 (severe), level 3(heavy), and level 4 (general). However, there is no unified standard on the level of internet public opinion early warning for major emergencies. According to different types of events, the generated network public opinion has a specific stage of development. Based on the existing literatures and the characteristics of the network, this paper divides the warning level into five levels respectively for level 1 (especially major warning), level 2 (major warning), level 3 (lager warning) level 4 (general warning) and level 5 (safety warning).

According to the CEI, the classification of different warning levels is manifested in Table 7.

**TABLE 7:** Classification Table of Network Public Opinion Warning Level

Warning level	Description	CEI value
1	Especially major warning	[0,20]
2	Major warning	(20,40]
3	Lager warning	(40,60]
4	General warning	(60,80]
5	Safety warning	(80,100]

### C. Early Warning Level Prediction Based on GA-BP Neural Network

For COVID-19 network public opinion, in addition to learn how to classify the early warning levels, more attention should be paid to how to predict the early warning levels. Therefore, following the classification of warning levels using the “CRITIC” method, an appropriate method for predicting warning levels needs to be established. In this study, the method of GA-BP neural network is selected.

Using GA-BP method, the number of layers and nodes of each layer of BP neural network algorithm should be determined first. In this study, a three-layer neural network structure with the input layer, hidden layer, and output layer is selected. Since there are 13 second-level indexes in the network public opinion early warning index system for major emergencies, the number of nodes in the input layer is set as 13. Because the warning level of network public opinion for major emergencies is divided into five levels, the number of nodes in the output layer is set as 5. In general, however, the number of hidden layer nodes has no definite value. In this case, it is indispensable to use the generally used calculation formula to determine the number of hidden layer nodes:

\begin{equation*} N=\sqrt {m+n} +a\tag{14}\end{equation*}

where, $N$ represents the number of hidden layer nodes; $m$ represents the number of nodes at the input layer, $m=13$; $n$ represents the number of nodes in the output layer, $n=5$; $a$ is a constant, between 1 and 10. Obviously, in order to determine the number of hidden layer nodes, the value of $a$ must be determined. Regarding this point, $a$ is traversed from 1-10, and the optimal value of $a$ is selected by comparing the prediction performance of BP neural network.

Before fitting BP neural network, we normalize all data and divide the data into two classes: training data and testing data. Most of the researchers used 70:30 ratio for divide data sets. It also depends on data characters, data size etc. In this study, we divide it into training and testing sets with the 80/20 split and use a stratified 5-fold cross validation. The R language has an add-on package named nnet that allows to create a neural network classifier. The prediction accuracy and error of the training and testing sets are demonstrated in Table 8.

**TABLE 8:** The Prediction Accuracy and Error of BP Neural Network Under Different $a$ Value

$a$	Training accuracy	Training error	Test accuracy	Test error
1	0.97	0.03	0.67	0.33
2	0.99	0.01	0.58	0.42
3	0.98	0.02	0.67	0.47
4	1.00	0.00	0.73	0.27
5	0.99	0.01	0.56	0.78
6	1.00	0.00	0.71	0.42
7	1.00	0.00	0.67	0.47
8	1.00	0.00	0.67	0.40
9	1.00	0.00	0.64	0.53
10	1.00	0.00	0.67	0.47

As presented in Table 8, the value with the best performance is when $a=4$. According to formula (14), the number of nodes N in the hidden layer is set to 8. Consequently, the structure of BP neural network consists of input layer with 13 nodes, hidden layer with 8 nodes and output layer with 5 nodes. Further, the initial weights and thresholds of BP neural network can be optimized by GA. According to the network structure of 13-8-5, the neural network has 13 * 8 + 8 * 5 = 144 initial weights and 8 + 5 = 13 initial thresholds, totaling 144 + 13 = 157 initial parameters. As a result, the length of the individual encoding of GA is 157. The best fitness value for a population is the smallest fitness value for any individual in the population. In this study, the fitness function $F$ is constructed with neural network prediction error:

\begin{equation*} F=\mathop \sum \limits _{i=1}^{K} \left |{ \hat {y}_{i}-y_{i} }\right |\tag{15}\end{equation*}

where $K$ represents the sample number of the training set; $\hat {y}_{i}$ denotes the predicted warning levels; $y_{i} $ represents the actual warning levels.

Subsequently, the genetic algorithm is used to minimize the initial fitness function $F$ and to generate high-quality solutions. Taking the optimal individual encoding as the initial weights and thresholds of BP neural network can increasingly improve the prediction ability of the neural network. The mcga function of the mcga package in the R language is used to implement machine coded genetic algorithms for real-valued optimization problems. The values of parameters for genetic algorithm model are shown in Table 9.

**TABLE 9:** The Specific Parameters of the Genetic Algorithm

Name of parameters	Parameter setting
Number of chromosomes	popsize=100
Number of parameters	chsize=157
The lower bound of the randomized initial population	minval=−10
The upper bound of the randomized initial population	maxval=10
The maximum number of generations	Maxiter=100
crossover probability	crossprob=0.2
mutation probability	mutateprob=0.1
fitness function	evalFunc=F

Given the above, GA-BP method is used to predict level of COVID-19 related activity on a network public opinion. In order to make full use of all the dataset, this study loops the program below a 100 times to obtain the average output.

**FIGURE 4.**
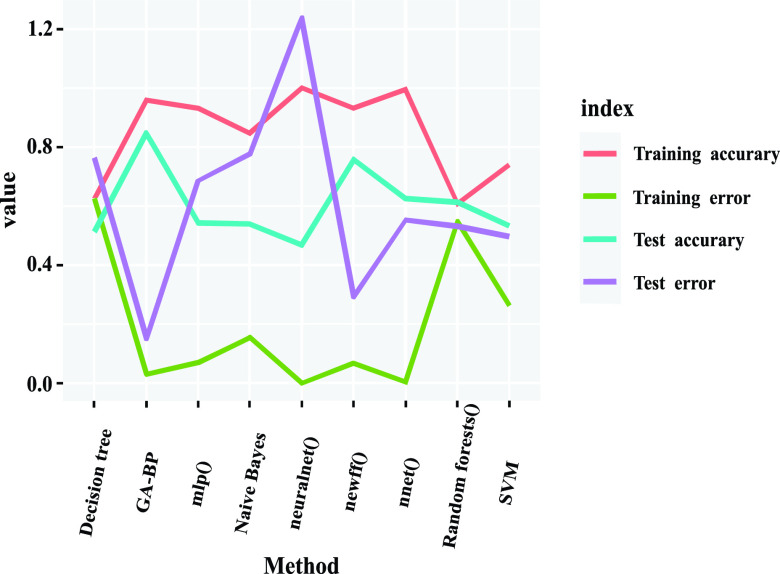
Prediction accuracy and error of different classification algorithms.

The accuracy of prediction of GA-BP model reaches 85%, and the average prediction error is only 0.15. It is suggestive that the accuracy of GA-BP neural network prediction model can reach the high and the error is extremely small. Nevertheless, the accuracy and feasibility of the network public opinion warning index system and warning model need to be further verified. So that five common machine learning classification algorithms are used, namely BP neural network, decision tree, random forest, naive Bayes and support vector machine. The predictive accuracy of the algorithms mentioned above are compared with GA-BP algorithm model to judge the feasibility of GA-BP model in network public opinion early warning of major emergencies. Besides, this study uses four packages (nnet, neuralnet, AMORE and RSNNS) in the R language to fit the BP neural network respectively and compare the performance of each package. There are two basic measures for evaluating early warning performance: predictive accuracy and mean square error. There are graphically shown in Figure 4.

Table 10 demonstrates that the GA-BP neural network model of network public opinion pre-warning is most suitable. Besides, the performance of BP neural network models fitted by functions in different R packages is also intensely different. The best is the BP neural network model fitted by the newff function in the AMORE package, with a prediction accuracy of 76% and an average prediction error of 0.29. The worst is the BP neural network model fitted by the neuralnet function in the neuralnet package, with less than 50% prediction accuracy and the highest average prediction error. Moreover, the prediction effect of the other four classification algorithms is not much different, but their performance is exceedingly lower than that of GA-BP neural network model. The Random forests classifier is the most effective, with an accuracy of 61% and an average prediction error of 0.53. The worst is the Decision tree classifier, with an accuracy rate of 51% and an average prediction error of 0.76.

**TABLE 10:** The Specific Calculation Results of Different Classification Algorithms

Classification algorithms		Training accuracy	Training error	Test accuracy	Test error
GA-BP neural network		0.96	0.03	0.85	0.15
BP neural network	nnet()	1.00	0.00	0.63	0.55
	neuralnet()	1.00	0.00	0.47	1.24
	mlp()	0.93	0.07	0.54	0.69
	newff()	0.93	0.07	0.76	0.29
SVM		0.74	0.26	0.53	0.50
Decision tree		0.62	0.63	0.51	0.76
Random forests		0.61	0.55	0.61	0.53
Naïve Bayes		0.85	0.15	0.54	0.78

As this research have shown, the BP neural network model optimized by GA is better than that all kinds of classification algorithm under the same conditions. Both prediction accuracy and prediction error, it demonstrates that the GA-BP neural network model of network public opinion pre-warning is more suitable comparing with the BP network. In fact, this method optimizes the structure of the BP neural network efficiently and overcomes a series of problems occurring in the BP neural network such as falls into the local minimum easily and bad computational stability. Moreover, the experimental results show that GA-BP neural network model makes the convergence rate faster.

## V. Conclusion

In this study, the early warning index system of network public opinion for major emergencies is constructed. The CRITIC method is used to determine the weight of each index and divide the warning level of each time point by calculating the comprehensive evaluation index. Finally, we use GA-BP neural network method to build the network public opinion level warning model of major emergencies. This study takes the internet public opinion of COVID-19, a major public health event, as an example of empirical analysis. The following conclusions can be drawn.

First, the network public opinion warning index system for major emergencies is constructed in this study includes 4 first-level indexes and 13 second-level indexes. The experimental results manifest that the early warning accuracy rate of GA-BP neural network algorithm is as high as 86%, indicating that these indexes possess a significant effect on the level of network public opinion. In other words, these indexes can be used to determine the level of internet public opinion. It demonstrates that the construction of the index system is reasonable. Therefore, these indexes in the index system can be used to carry out public opinion early warning management work promptly.

Second, the BP neural network based on Genetic Algorithm is used to establish a network public opinion early warning model, and compares the performance of several shared classification algorithms: BP neural network, decision tree, random forest, naive Bayes, and support vector machine. The experimental results demonstrate that GA-BP neural network has the best prediction accuracy and the smallest mean square error. It can be concluded that the GA-BP neural network model is feasible, effective, and accurate in internet public opinion early warning.

Third, four packages fitting BP neural network in the R language are used for comparison. The results testify that the prediction accuracy and mean square error of the model established by the newff function in the AMORE package reach the optimal level.

Ultimately, the following suggestions are put forward for the early warning management of network public opinion under the influence of major emergencies. First of all, it is indispensable to exert the influence of media interaction platforms and control the fermenting degree of internet public opinion under major emergencies with the aid of the media strength. Weibo, Baidu, and other media with more active users must give play to the self-discipline of their platforms. They can establish a network environment management security group and set up constraints on those who publish or disseminate information to ensure the discipline of the network environment. Secondly, users who publish false statements or false news in cyberspace must be punished accordingly. It can be observed from the index system that the influence of Weibo users and the authenticity of public opinion both affect the public opinion early warning level. Therefore, netizens should devote themselves to improve the correctness and self-discipline of their speech in the face of major emergencies. Finally, if scientific judgment and research are to be carried out, it is essential for the government to adopt advanced network public opinion early warning technology to timely discover the development trend of public opinion.

In today’s data-driven environment, with so much information out there, future research should focus on the use of big data platforms to automatically collect network public opinion information and adopt advanced technology for processing. Only in this way can we quickly grasp the information of internet public opinion and effectively improve the efficiency of early warning.
